# Inhibition and Formation of Amyloid Fibrils in the Bulk and at the Interface of Biomolecular Condensates

**DOI:** 10.1002/anie.202515409

**Published:** 2026-06-26

**Authors:** Marcell Papp, Chiara Morelli, Sarah Khawaja, Paolo Arosio

**Affiliations:** ^1^ ETH Zürich, Department of Chemical and Applied Biosciences Institute for Chemical and Bioengineering Zürich Switzerland

**Keywords:** amyloid formation, biomolecular condensates, inhibition and acceleration, interface, protein aggregation

## Abstract

Cells can form open compartments, known as biomolecular condensates, which possess distinct environments and concentrations compared to their surroundings. These biomolecular condensates can modulate biochemical processes, including protein aggregation. Notably, they have been reported to both accelerate and inhibit protein aggregation. Since protein aggregation is often associated with pathological conditions like neurodegenerative diseases, it is crucial to understand the molecular mechanisms underlying the interplay between phase separation and fibril formation. In this review, we discuss how, contrary to intuition, aggregation within the bulk of condensates can be inhibited rather than promoted, even in the presence of elevated local protein concentration. However, biomolecular condensates can still facilitate fibril formation by generating an interface between the dense and dilute phases, where molecular and mesoscale properties are optimal for the nucleation of protein aggregation.

## Graphical abstract

In this review, we discuss how biomolecular condensates can inhibit amyloid aggregation in their interior, while still facilitating fibril formation at the interface between the dense and dilute phases, where molecular and mesoscale properties are likely optimal to promote protein aggregation.

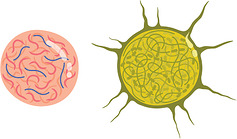

## 1 Introduction

The formation of insoluble protein aggregates known as amyloid fibrils is a hallmark of many neurodegenerative diseases, including frontotemporal dementia (FTD), amyotrophic lateral sclerosis (ALS), Alzheimer's disease (AD), and Parkinson's disease (PD) [1, 2, 3, 4, 5]. Amyloids have a distinctive cross‐β structure and are extremely thermostable [6]. The solution‐to‐solid transition underlying amyloid formation is typically described with a multi‐step mechanism involving nucleation and elongation events [7], where incorrect or partial folding of proteins can lead to aggregation‐prone monomers, which associate to form various oligomeric species and nucleate into amyloid fibrils [8]. Once a nucleus is formed, fibrils elongate rapidly through self‐templated growth [9].

In some cases, the formation of pathological amyloids is associated to the recruitment of the protein into membraneless organelles (MLOs), which are biomolecular condensates formed through phase separation in the cell [10, 11, 12]. MLOs play an important role in many biological processes, for instance, by functioning as quality control systems that sequester misfolded proteins [13] or as reaction centers that modulate the local concentration of enzymes and their substrates [14, 15]. Some MLOs, such as the nucleolus [16] are constitutively present, while others assemble and disassemble dynamically. Under certain conditions, some MLOs lose their dynamic nature and harden [17], or mature into irreversible protein aggregates [18, 19, 20, 21, 22, 23], a process associated with disorders. Indeed, many disease‐related amyloid‐forming proteins, including FUS, tau, α‐synuclein, hnRNPA1, TIA‐1, and TDP‐43, have been shown to undergo phase separation in vitro as an intermediate step before their conversion into fibrils [24]. Phase separation and amyloid formation can thus be seen as distinct phase transitions [25] that obey Ostwald's rule of stages, which anticipates the formation of the thermodynamically less favored but kinetically more available liquid state, followed by a transition to the more stable amyloid state [26]. Because the rate of amyloid formation typically follows a power‐law dependence on monomer concentration [7], an obvious mechanism by which MLOs could accelerate the formation of protein aggregates is the increase in local protein concentration, which can reach several orders of magnitude higher than the surrounding dilute phase. Monomer concentration is not, however, the only determinant of aggregation rates, and factors such as protein conformation and effective diffusivity can strongly impact nucleation and elongation rate constants. The condensate interior can thus influence amyloid formation through various mechanisms [27], including, as we will see below, those that inhibit rather than promote aggregation.

Another risk factor for protein aggregation is the condensate boundary, which is a unique microenvironment with physicochemical properties distinct from both the dense and dilute phase. Interfacial properties can promote aggregation and a growing list of proteins with different sequences and architectures have shown accelerated amyloid formation at the condensate interface [22, 28, 29, 30, 31, 32]. This is reminiscent of surface‐induced protein aggregation, which has been observed at solid–liquid, air–water, and oil–water interfaces in many different fields, from in vitro studies of amyloids [33, 34, 35, 36] to manufacturing and development of protein drugs [37, 38]. Despite this, the molecular mechanisms that determine protein aggregation rates at the condensate interface can be drastically different and remain poorly understood.

In this review, we discuss how the bulk interior of condensates can affect amyloid fibril formation, focusing on the often overlooked inhibitory effects of this environment. Moreover, we discuss possible ways in which the condensate interface can influence amyloid fibril formation, distinguishing between client‐scaffold systems and single‐scaffold condensates.

## 2 Inhibition of Aggregation Inside Biomolecular Condensates

**FIGURE 1:**
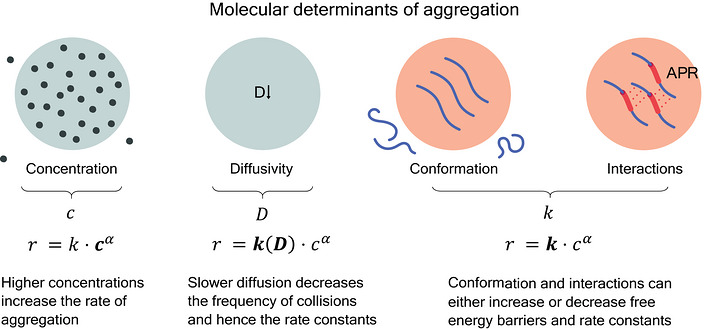
Various molecular determinants can affect the rate of aggregation (r) in the bulk of condensates, including diffusivity, conformations and interactions between aggregation‐protein regions (APRs) and other components of the condensates.

The condensate phase differs markedly from the dilute phase in both composition and density, representing a distinct physicochemical environment that can modulate protein aggregation in various ways. The molecular determinants include concentration, diffusion, protein conformation, and interactions with condensate components, as recently discussed in [27]. The potential effects of these molecular determinants on the rate of aggregation are summarized in Figure 1. In brief, aggregation rates often follow a power law dependence on protein concentration, characterized by an exponent, which is typically larger than one. Therefore, higher protein concentrations accelerate aggregation. The aggregation rate is also dependent on a kinetic rate constant, which is linked to the frequency of the collisions and the free energy barrier of the process. While molecular diffusivities affect collisions, the conformational landscape of the protein and its interactions with the surroundings modulate the energy barrier. Some of these effects can reduce aggregation rates despite the higher local monomer concentration. For instance, condensates can exhibit viscoelastic behavior that can strongly decrease diffusivity [39], causing fast reactions, such as fibril elongation, to become limited by mass transfer [40].

Furthermore, the high concentration in the dense phase can promote interactions between the aggregation‐prone proteins and other components present in the condensate, which can outcompete homotypic interactions required for amyloid formation. This phenomenon, termed heterotypic buffering [41], can occur through non‐specific interactions between the client and the scaffold proteins, or through the recruitment of specific proteins, such as chaperones, that inhibit the liquid‐to‐solid transition.

Such interactions, along with a plethora of other factors in the drastically different milieu within condensates, can also modify the conformational landscape of biomolecules [42, 43]. Since protein conformation has a crucial impact on amyloid fibril formation, in particular nucleation, changes in the relative abundance or stability of aggregation‐prone conformations within the condensate can modulate aggregation kinetics.

In the following, we distinguish between client‐scaffold systems, where the aggregating protein is distinct from the condensate‐forming protein, and single‐scaffold condensates, where the aggregating protein is the primary protein component of the condensate.

### 2.1 Client‐Scaffold Systems

**FIGURE 2:**
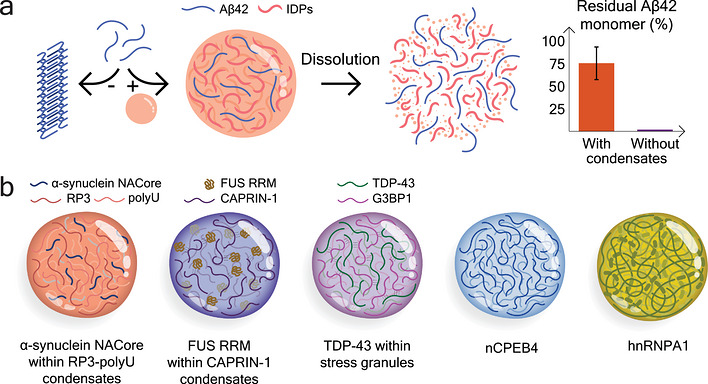
Examples of client‐scaffold and single‐scaffold systems, in which aggregation is inhibited in the bulk of condensates. Examples are taken from: top row [44], bottom row refs. [23, 28, 45, 46, 47].

One example of condensate‐related suppression of protein aggregation is the highly aggregation‐prone peptide, Aβ42, associated with Alzheimer's disease, whose aggregation was inhibited upon recruitment into condensates formed by a variety of scaffold proteins (Figure 2a) [44]. The Aβ42 peptide remained monomeric within the biomolecular condensates despite its local millimolar concentration. In contrast, the peptide aggregated within hours at nanomolar concentrations in the absence of biomolecular condensates. Another example is represented by the protein α‐synuclein, associated with Parkinson's disease, inside coacervates formed by RP3 and polyU (Figure 2b) [28].

While in these examples the exact molecular mechanism of inhibition remains to be fully elucidated, these initial observations have been followed by other studies, which determined how the molecular determinants summarized in Figure 1 affected aggregation.

For instance, it has been shown that the aggregation‐prone RNA‐recognition motif of FUS is upconcentrated within liquid condensates of the protein CAPRIN‐1, where the monomeric protein unfolds but does not aggregate. The environment of the CAPRIN‐1 condensate inhibits the formation of aggregates of the FUS RRM through multiple heterotypic intermolecular interactions, which have been unraveled at the residue level by solution NMR measurements [45].

An additional example is TDP‐43, which, under stress conditions, mislocalizes to the cytoplasm and accumulates within stress granules. Recent studies have shown that oxidative stress induces partial unfolding of its RNA‐recognition motif 1 (RRM1) and promotes disulfide bond formation between cysteine residues [46]. This oxidation event is accompanied by demixing of TDP‐43 from other stress granule components, thereby facilitating its aggregation [46]. This study illustrates how the failure of heterotypic buffering of TDP‐43 inside stress granules during oxidative stress may be a key mechanism driving its aggregation [46]. This liquid‐to‐solid phase transition can impair the reversibility of stress granules, preventing TDP‐43 from returning to its nuclear functions after stress cessation. Such dysregulation is potentially linked to neurodegenerative diseases such as ALS and FTD.

### 2.2 Single‐Scaffold Systems

Heterotypic buffering can also result from intermolecular interactions between two distinct regions of proteins of the same type (Figure 2b). For example, irreversible aggregation of CPEB4, an RNA‐binding protein associated with autism spectrum disorder, is inhibited by interactions between microexon 4, an 8 residue‐long exon rich in arginines, and a His‐rich region that is prone to aggregation [47].

Interactions within the dense phase can also promote the formation of strong networks that compete with fibril formation. For instance, the intrinsically disordered region of the ALS‐linked nuclear RNA‐binding protein, hnRNPA1A forms amyloid fibrils at the condensate interface following phase separation [22, 48] but its aggregation is actually inhibited in the interior of the condensates and fibrils grow by recruiting monomer in solution [23]. Increasing the strength of the network inside the dense phase by strengthening the sticker–sticker interactions competes with amyloid aggregation [23]. Moreover, aging of condensates can lead to the formation of non‐fibrillar, β‐sheet‐containing solids, creating a barrier for the conversion into amyloid fibrils [17].

These examples show that despite the high local concentration, the bulk of biomolecular condensates can serve as a protective environment against protein aggregation, rather than triggering fibril formation. This can have biological relevance for biomolecular condensates involved in quality control systems, such as the nucleolus, which can sequester misfolded proteins and prevent their aberrant aggregation [13]. Hence, it is crucial to consider multiple, often correlated molecular effects that govern the promotion or inhibition of amyloid aggregation in addition to local protein concentration (Figure 1).

## 3 Promotion of Aggregation at the Interface of Biomolecular Condensates

While aggregation in the bulk of the condensates may be inhibited, numerous examples show the promotion of fibril formation via liquid‐to‐solid transition of biomolecular condensates at the interface between the two coexisting phases [22, 28, 29, 30, 31, 32, 49].

**FIGURE 3:**
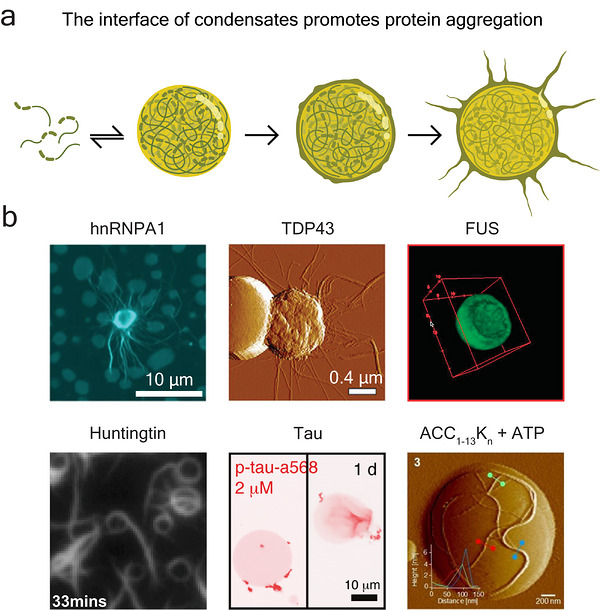
(a) The interface of biomolecular condensates promotes protein aggregation. (b) Various experimental examples of interfacial aggregation of disease‐associated proteins, such as hnRNPA1 [22] TDP‐43 [31], FUS [30], Huntingtin [53], Tau [54]; and an artificial fusion protein ${\mathrm{ACC}}_{1-13}K_{n}$ with ATP [32]. Images were taken from: Top left: ref. [22], used under CC BY 4.0 license. Top middle: ref. [31], used under CC BY 4.0 license. Top right: ref. [30], used under CC BY 4.0 license. Bottom left: ref. [53], used under CC BY 4.0 license. Bottom middle: ref. [54], used under CC BY 4.0 license. Bottom right: ref. [32]. Reprinted with permission. Copyright 2023 American Chemical Society.

For instance, although the aggregation of hnRNPA1 is inhibited in the bulk of condensates [23], it is promoted at the interface between the dense and dilute phase (Figure 3a) [22, 29], This behavior has been observed with the intrinsically disordered region of the protein [22], as well as with the full length protein, both in the absence and presence of RNA [29].

Similarly to hnRNPA1, other proteins associated with ALS and FTD, such as FUS and TDP‐43, also formed solid structures at the interface or star‐shaped aggregates [31, 50] that originated from the interface [30, 51].

For TDP‐43, following liquid–liquid phase separation, AFM images showed that the fibrils originate from the condensates, suggesting an important role of the condensate interface in initiating the aggregation of TDP‐43 (Figure 3b) [31]. Moreover, the prion‐like domain of the protein was shown to be sufficient to form condensates and trigger the formation of amlyoids at the condensate interface, as shown by confocal microscopy [52].

For another ALS‐associated protein, FUS, the interface of the condensates transitioned from a liquid state to a β‐rich solid shell, while the interior of the condensates remained liquid‐like and could be dissolved by increasing ionic strength [30] (Figure 3b). As the condensates aged, the solid shell became progressively thicker, indicating that gel formation propagated inward. Over extended incubation times, filamentous structures also formed around the condensates [30].

In addition to other natural disease‐related sequences, including Huntingtin [53] and Tau [54], artificial constructs comprising a phase‐separating poly‐lysine tail and a region prone to aggregation also underwent a liquid to solid transition, where fibrils appeared at the phase boundary (Figure 3b) [32].

In the following sections, we look at potential mechanisms through which aggregation is promoted at condensate surfaces both in client‐scaffold systems and single‐scaffold condensates.

### 3.1 Possible Mechanisms of Promotion of Aggregation at the Condensate Interface

#### 3.1.1 Client‐Scaffold Systems

**FIGURE 4:**
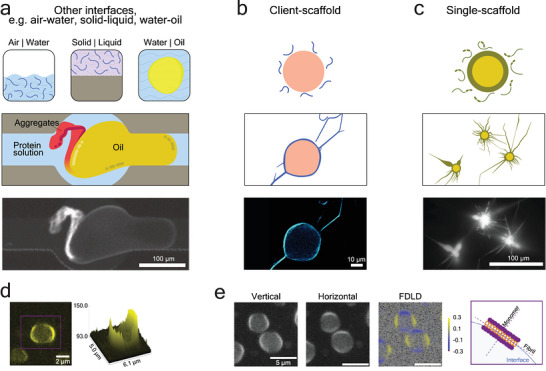
(a) Interfaces, such as air–water and oil–water interfaces, are well known risk factors for protein aggregation. Image taken from ref [38], used under CC BY 4.0 license. (b) Protein aggregation at the interface of client‐scaffold condensates (c) and single‐scaffold condensates. Images were taken from refs. [22, 44], used under CC BY 4.0 license. (d) Fibrils of hnRNPA1 emerge at the condensate interface and (e) are oriented parallel to the condensate surface. Images were taken from ref [29], used under CC BY 4.0 license.

The presence of interfaces has been a well‐known risk factor for protein aggregation (Figure 4a). The formation of aggregates in a solution without impurities or surfaces (homogeneous aggregation) depends on rare events and can therefore be a slow process, especially when the protein concentration is low. In contrast, heterogeneous aggregation, which occurs at interfaces, can significantly accelerate the assembly of protein monomers through various mechanisms [55]. Heterogeneous aggregation of amyloid fibrils involves a cascade of elementary steps, including adsorption, conformational change, nucleation and growth, and release of aggregates from the surface [55]. Protein adsorption can promote aggregation by locally increasing protein concentration, as long as the binding interaction is not so strong to suppress translational mobility of the monomers and the final detachment of the aggregates [56]. Moreover, binding to surfaces can also induce conformational changes of the monomers or conversion of oligomeric species into amyloid nuclei that lower the overall free energy barrier of nucleation [55]. Accordingly, adsorption to air–water, solid–liquid, or oil–liquid surfaces were shown to induce the aggregation of amyloidogenic and therapeutic proteins (Figure 4a) [33, 34, 35, 36, 37, 38, 56].

The liquid–liquid interface of biomolecular condensates may have analogous effects on the formation of amyloid aggregates, especially when the main condensate‐forming protein (the scaffold) differs from the aggregating species (the client) (Figure 4b). Client monomers can preferentially localize at the interface of biomolecular condensates. In general, this does not necessarily involve specific binding to the condensate‐forming scaffold molecules. Interfacial accumulation can occur when distinct parts of a client protein have different affinities for the dilute and dense phases, creating a hydrophobic moment [57] analogous to the electrostatic dipole moment. This moment can originate from spatially distant hydrophilic and hydrophobic surface patches [58] or separate domains of surfactant‐like proteins [59]. In some cases, clients with little affinity for the dense phase weakly localize at the interface to reduce the interfacial tension of condensates [60]. In all cases, the adsorbed client proteins locally increase their concentration while possibly changing their configuration, which may promote amyloid formation. For instance, Parkinson's disease‐related protein α‐synuclein adsorbed and aggregated at the interface of polyK/polyE condensates [28]. The adsorption of α‐synuclein at the interface of complex coacervates involves weak interactions with scaffold molecules, which are mediated by electrostatic attraction between the positively charged protein and negatively charged condensate interface [61].

Once the client molecules are adsorbed, the promotion of aggregation can involve a conformational change of the interfacial monomers, following mechanisms similar to those observed with lipid vesicles and the air–water interface [33, 62].

Proteins that localize at the interface can also retain their rotational degrees of freedom [63], where their orientation can create molecular order. Since amyloid fibrils are highly structured, semi‐crystalline species [64], their nucleation can also be facilitated by this long‐range symmetry in the molecular orientation at the condensate interface and the formation of a template for the ordered assembly of monomers [65, 66].

We note that aggregation at the interface does not necessarily involve preferential partitioning at the interface. In some cases, indeed, client monomers are uniformly distributed within the condensates, similar to single‐scaffold condensates, although aggregation is still promoted at the interface [28, 44].

#### 3.1.2 Single‐Scaffold Condensates

The case of condensates, which consist of a single scaffold that is also prone to aggregation, is more complex. Future studies are needed to elucidate the detailed mechanisms that promote aggregation and the peculiar properties of the interface of the condensates. In the following, some considerations are presented based on recent evidence.

If the condensate has a strong internal network, and the exchange rate of monomers between the two phases is slow, monomers diffusing from the dilute phase could transiently adsorb and accumulate at the interface, promoting aggregation. In this case, aggregating monomers are likely to originate from the dilute phase, and the molecular effects that influence aggregation in single‐scaffold condensates can be similar to client‐scaffold systems, as summarized in Figure 4b.

**FIGURE 5:**
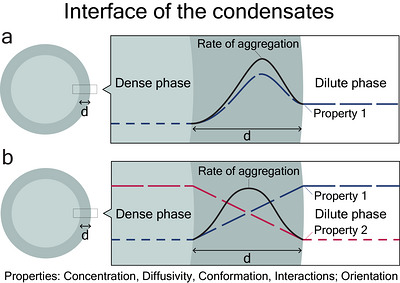
Various scenarios can explain how molecular determinants at the interface (such as concentration, diffusivity, conformation, interactions, and orientation) may promote protein aggregation. (a) Some of these properties may exhibit higher values at the interface compared to both the dilute and dense phases, or (b) different properties may follow monotonic gradients with opposite signs, resulting in intermediate values between the two phases. The combined effects of these properties can synergistically increase the aggregation rate. For example, the interface may display intermediate diffusivity (property 1) and concentration (property 2), leading to enhanced aggregation compared to the bulk (where diffusivity is too low) and the dilute phase (where concentration is too low).

In other cases, interfacial scaffold proteins can create a unique bridging region between the coexisting bulk phases, where they can exhibit peculiar properties (Figure 5a).

For instance, cryogenic electron microscopy experiments showed that the protein concentration of the RGG domain of Laf1 is higher at the interface of their condensates [67]. Biomolecular condensates therefore might have a core–shell structure with a denser shell close to the interface. In another example, Raman spectroscopy showed a higher concentration of the low‐complexity domain of hnRNPA1 at the interface of the condensates [22].

Despite the different mechanisms behind the high local concentration, in all cases, the increased abundance of proteins could contribute to faster interfacial aggregation in the case of amyloidogenic scaffold proteins in the same way as for client molecules.

In addition to concentration, diffusivity can also differ at the interface. The temporary diffusion coefficient of scaffold molecules was shown to increase at the phase boundary relative to both bulk phases in coarse‐grained molecular dynamics simulations of the low‐complexity domain of FUS and DDX4 [68]. Furthermore, anisotropic molecular motions were measured at the interface of poly‐adenine (poly‐rA)–polyethylene glycol (PEG) condensates [69]. Single‐molecule tracking indicated that poly‐rA molecules diffused considerably faster parallel to the interface compared to the perpendicular direction. These examples demonstrate that molecular mobility can vary significantly at the phase boundary. In particular, increased diffusivities could enhance aggregation rates in contrast to the interior of the condensate, where mass transfer limitations are expected.

The molecular conformation could potentially change in the case of interfacial scaffold molecules as well. Monte‐Carlo simulations of the low‐complexity domain of hnRNPA1 predicted elongated conformations at the interface of condensates [70], which may drive β‐sheet formation. In addition to the higher fluorescence intensity using non‐polarized light (Figure 4d), fluorescence‐detected linear dichroism imaging showed that fibrils of the full‐length hnRNPA1 are oriented parallel to the interface, where the amyloid‐forming monomers are perpendicular to the phase boundary (Figure 4e) [29]. Cryo‐EM data are also consistent with an elongated conformation and tighter packing of monomers at the interface of the RGG domain of Laf1 [67], which could also be applicable to amyloid‐forming species. The protein conformation is affected by multiple interactions between the scaffold molecules and the solvent. The concentration and activity of water crucially affect the hydration of both the side chains and the backbone of proteins [71, 72], which plays a major role in amyloid formation [73, 74, 75, 76]. The altered hydrogen‐bond network and solvation properties of interfacial molecules could modulate the conformation and, thus, the aggregation propensity.

Moreover, asymmetric ion partitioning between the phases can lead to interphase electric potentials, creating a double layer at the condensate interface [77, 78, 79]. Protein chains with a substantial electric dipole moment might prefer elongation and perpendicular orientation with respect to the interface due to this emerging electrostatic field at the phase boundary. In this way, interfaces might promote extended, aggregation‐prone conformations [80, 81, 82], lowering the free energy barrier of nucleation.

The previous examples have shown how the interface is a unique connecting region between the bulk of the dilute and dense phases and that, in this area, molecular determinants of protein aggregation can exhibit maxima or minima compared to the surrounding phase (Figure 5a), for example, in terms of concentration [22, 67], diffusivity [68], or conformation [70], We note, however, that the interface could promote aggregation even when no distinct minimum or maximum values of these properties are present. Even monotonous gradients of molecular properties across the interface could lead to an ideal trade‐off between the different molecular determinants shown in Figure 2 (Figure 5b), including protein concentration, ion concentration, water concentration, solvation of the backbone and side chains, as well as molecular mobility and conformation. These properties often interact in complex ways, exerting opposing effects on the rate of amyloid formation (Figure 5). For instance, the balance between a decreasing diffusion coefficient, conformational mobility, and higher protein concentration could lead to an optimal environment for amyloid formation within the interfacial region, where both concentration and mobility are sufficiently high to facilitate aggregation (Figure 5). Overall, predicting the combined effects of these gradients is challenging due to their complexity and the non‐linearity of their individual contributions to the aggregation rate. Consequently, even in the presence of monotonous gradients, the overall impact of opposing gradients may not be straightforward but could result in a maximum rate of aggregation.

## 4 Role of Condensate Interface Beyond Amyloid Formation

The peculiar effects at the interface of condensates discussed in the previous paragraphs may underlie not only the interplay between phase separation and amyloid formation but also the role of condensates in modulating other biochemical reactions, such as enzymatic reactions [15]. Indeed, in addition to local concentrations, all molecular and mesoscale properties within condensates affect binding events and kinetic rate constants. Condensates can therefore act as “effective solvents,” and this effect can significantly modulate biochemical reactions in addition to changes in concentration and mass action law [83, 84, 85]. The interface of the condensates could behave as a distinct environment with properties that are intermediate between the bulk of condensates and the dilute phase. These considerations are important not only to understand possible roles of condensates in modulating cellular functions but also for applications in metabolic engineering [86]. For instance, it is emerging that the size of condensates can strongly impact the effect on enzymatic reactions, with the strongest acceleration of reactions observed when the size is in the nanometer range [84, 87]. This observation can be explained by the presence of mass transfer limitations, which have been both experimentally measured and predicted by simulations [84, 87]. However, the role of the interface could further contribute to this size effect.

## 5 Conclusion

The role of phase separation in the formation of pathogenic amyloid fibrils has remained elusive. While the local concentration of proteins drastically increases within biomolecular condensates, other molecular factors simultaneously affect aberrant protein aggregation, such as molecular mobility, protein conformation, and orientation. Several experimental examples have suggested an inhibitory role for condensation as opposed to triggering amyloid formation. However, biomolecular condensates also introduce a liquid–liquid interface between the coexisiting phases, which can promote the formation of amyloid aggregates. The interface between biomolecular condensates and the dilute phase shares analogies with other types of surfaces, and can explain enhanced interfacial amyloid formation in the case of the aggregation of client proteins at host condensates. Additionally, the condensate‐forming scaffold proteins in the interfacial region experience the proximity of both the solvent and the protein‐rich phase, which could significantly alter the molecular determinants of aggregation kinetics at the phase boundary.

Advances in experimental characterization techniques are required to fully understand the interfacial properties of biomolecular condensates. Currently, interfacial aggregation has been analyzed using a combination of indirect bulk methods, such as Thioflavin T kinetic assays, and optical and spectroscopic techniques, including confocal microscopy and 3D scanning [30], spatial dynamic mapping [30], fluorescence recovery after photobleaching [49], and Raman spectroscopy [51]. Emerging methods capable of directly quantifying properties such as concentration, interactions, and diffusivity at the interface [69, 88] will be crucial for dissecting the individual effects of the molecular determinants summarized in Figure 1.

In this context, approaches capable of specifically modulating the interface without impacting bulk properties can reveal very useful results [89].

## Conflicts of Interest

The authors declare no conflicts of interest.

## Data Availability

Data sharing is not applicable to this article as no new data were created or analyzed in this study.
